# ALKBH5 Improves the Epithelial Cell Tight Junctions to Inhibit *Escherichia coli*-Induced Mastitis

**DOI:** 10.3390/cells14070521

**Published:** 2025-04-01

**Authors:** Xuan Wu, Haojun Xu, Yongchong Peng, Ruikai Zhang, Yanjun Hu, Aizhen Guo, Changmin Hu

**Affiliations:** 1 Department of Clinical Veterinary Medicine, College of Veterinary Medicine, Huazhong Agricultural University, Wuhan 430070, China; wuxuan@webmail.hzau.edu.cn (X.W.); haojun.xu@duke.edu (H.X.); 2 National Key Laboratory of Agricultural Microbiology, Huazhong Agricultural University, Wuhan 430070, China; ycpeng@mail.hzau.edu.cn (Y.P.); huyanjun@webmail.hzau.edu.cn (Y.H.); aizhen@mail.hzau.edu.cn (A.G.); 3 Department of Preventive Veterinary Medicine, College of Veterinary Medicine, Huazhong Agricultural University, Wuhan 430070, China; 4 Veterinary Pathology Laboratory, College of Veterinary Medicine, Huazhong Agricultural University, Wuhan 430070, China; zhangrk@webmail.hzau.edu.cn; 5 Hubei Hongshan Laboratory, Huazhong Agricultural University, Wuhan 430070, China; 6 The Veterinary Teaching Hospital, College of Veterinary Medicine, Huazhong Agricultural University, Wuhan 430070, China

**Keywords:** ALKBH5, tight junction, *E. coli*, mastitis, p65, TJP1

## Abstract

Mastitis poses a severe threat to the global cattle industry, causing huge economic losses. Environmental mastitis is mainly induced by *Escherichia coli* (*E. coli*), and the current treatment is still using antibiotics, with problems such as drug resistance and food safety. ALKBH5 is an RNA m^6^A demethylase that plays an important role in various biological processes, while p65 is a key regulator of inflammatory responses. Therefore, studying the interaction between ALKBH5 and p65 in protecting the mammary epithelial barrier provides new insights into the pathogenesis of mastitis. This study revealed that *E. coli*-induced acute inflammation activated the NF-κB/p65 signaling pathway and disrupted mammary epithelial cell tight junctions. Knockdown of ALKBH5 promoted p65 phosphorylation and inhibited the expressions of the tight junction proteins TJP1, CDH1, and OCLN. Furthermore, motif analysis, CHIP-PCR, and dual luciferase assay confirmed that phosphorylated p65 inhibited *TJP1* promoter activity, thereby inhibiting TJP1 expression. In addition, the mouse experiment further demonstrated that knockdown of ALKBH5 aggravated *E. coli*-induced acute mastitis and epithelial cell tight junction disruption, and promoted *E. coli* invasion and proliferation. Significantly, this study is the first to demonstrate the details of the interaction between p65 and TJP1 and to declare the molecular mechanism of ALKBH5 in improving the cell tight junction, which lays a potential target and theoretical foundation for the treatment of mastitis and other infectious diseases.

## 1. Introduction

Bacteria are the primary causative organisms of mastitis in dairy cattle [1]. More than 150 Gram-positive and -negative bacteria have been determined as mastitis pathogens [2], and *Escherichia coli* and *Staphylococcus aureus* are the most common pathogenic bacteria although the incidence of mastitis varies globally [3,4]. *E. coli*-induced mastitis is the leading cause of premature value loss in dairy cattle and poses a serious challenge to public safety. *E. coli* causes acute inflammatory damage, even when the pathogen breaks through the mammary epithelial barrier, and causes systemic infection, resulting in acute death of dairy cattle. Current treatment focuses on antibiotics, which are not effective and frequently cause the emergence of drug-resistant strains, urging researchers to establish more effective mastitis treatment strategies [5,6,7].

Epithelial cells in dairy cattle serve as the primary defense mechanism for innate immunity in the mammary gland [8]. Cell tight junctions form the structural basis of the epithelial cell barrier by creating adjacent cell membranes that tightly adhere and close the intercellular space, thereby resisting pathogen invasion [9]. Bacterial infection of the mammary gland in dairy cattle rapidly activates the innate immune and inflammatory responses [10], and further causes epithelial cell tight junction disruption and barrier damage [11,12,13,14]. Therefore, protecting the epithelial barrier function by improving cell tight junctions and thus resisting pathogen invasion is a new method for treating mastitis.

N^6^-methyladenosine (m^6^A) modification is the most common epigenetic modification on mammalian RNA [15,16]. Our previous study indicated that the m^6^A total amount was highly increased in the bovine mammary epithelial cells in *E. coli*-induced mastitis [17], so down-regulation of m^6^A might be a breakthrough for the treatment of mastitis. AlkB homolog 5 (ALKBH5), a demethylase of m^6^A, plays an important regulatory role in physiological and pathological processes [18,19]. One study found that ALKBH5 enhances neutrophil migration, thereby increasing cellular innate defense against bacteria [20]. Another study revealed that ALKBH5 can regulate cellular metabolic processes in macrophages to influence host–pathogen interactions [21]. Furthermore, in bovine mastitis, ALKBH5 regulated the stability of LncRNA4191 and inhibited *E. coli*-induced apoptosis of bovine mammary epithelial cells [17]. However, whether ALKBH5 promotes mammary epithelial cell resistance to bacterial invasion and protects the mammary epithelial barrier is unknown. Therefore, it is significant to explore the regulatory mechanism of ALKBH5 on epithelial cell tight junctions and mastitis, which is expected to be a new target for efficient bovine mastitis treatment.

In this study, we investigated the disruption of epithelial cell tight junctions in *E. coli*-induced acute mastitis in the bovine mammary epithelial cell model and the mice model. In addition, we explored the regulation of ALKBH5 on mastitis and cell tight junctions by knocking down ALKBH5. Furthermore, we studied the molecular mechanism by which ALKBH5 regulates the TJP1 expression via p65 and protects cell tight junctions. Overall, we found that ALKBH5 could be a potential target for bovine mastitis treatment by enhancing cell tight junctions and epithelial barrier.

## 2. Materials and Methods

### 2.1. Cell Line, Bacteria, and Mouse

Prof. Mark Hanigan (Virginia Tech University, Blacksburg, VA, USA) provided bovine mammary epithelial cells (MAC-T), cultured with our previous method [22]. Prof. Aizhen Guo (Huazhong Agricultural University, Wuhan, China) supplied human embryonic kidney cells (293T), cultured with DMEM/HIGH GLUCOSE (Cytiva, Marlborough, MA, USA) and 10% fetal bovine serum (ExCell Bio, Shanghai, China).

Prof. Xiangru Wang (Huazhong Agricultural University, Wuhan, China) donated *Escherichia coli* (ATCC 25922). Bioluminescent *E. coli* was constructed by placing *E. coli* (ATCC 25922) in the sensory state with CaCl_2_ and then transforming the pAKLUX1 plasmid (MIAOLING BIOLOGY, Wuhan, China) with heat shock.

Huazhong Agricultural University’s Laboratory Animal Center supplied 7-week-old KM mice, including 15 males and 30 females, weighing 35–45 g. The Laboratory Animal Ethics Approval of Huazhong Agricultural University approved the experiment (Approval No.: HZAUMO-2023-0341).

### 2.2. Gene Knockdown

Cells were inoculated into 12-well plates and cultured to a density of 70–80%. The siRNA of 50 nM was mixed with jetPRIME Transfection Buffer (PolyPlus, Illkirch, France) of 100 μL, then jetPRIME Transfection Reagent of 1 μL was added. The mixture was added to the 12-well plates and incubated for 24 h after placing at 25 °C for 10 min. The siRNA sequences are shown in Table S1.

### 2.3. Cell Adhesion Assay

The adhesion ability of MAC-T cells was detected with a cell adhesion assay kit (BestBio, Shanghai, China). Briefly, MAC-T cells were seeded onto a 96-well plate pre-coated with the reagents provided in the kit and incubated for 1 h at 37 °C to allow cell adhesion. Non-adherent cells were removed by gentle washing with PBS (Cytiva, Marlborough, MA, USA), then stained with the red fluorescent dye following the kit’s instructions, and incubated at 37 °C for 3 h. The adhesion assay was observed with the PE Opera Phenix (PerkinElmer, Waltham, MA, USA).

### 2.4. CHIP

A CHIP assay kit (Millipore, Billerica, MA, USA) was used to perform the CHIP assay. The cells underwent a 10-minute formaldehyde treatment, followed by crosslinking termination using glycine. The cells were lysed, sonicated, and the supernatant centrifuged as input. Immunoprecipitation was conducted with the antibody of p65 and IgG as a control. DNA was eluted from the precipitate, and PCR was conducted to detect the *TJP1* gene at the p65 and *TJP1* interaction sites (GGGAAATCCC). The primer sequences used were forward 5′-CATAGTAGTAAGCCTTTCTTCTGG-3′ and reverse 5′-ACCGCATGGGACTGTAGTCTG-3′. Table S2 shows the antibodies used.

### 2.5. Dual-Luciferase Assay

Promoter activity was detected with 293T cells that were cotransfected with PRL-TK as an internal reference and PGL3-Basic as a reporter plasmid. The promoters to be investigated included the *TJP1* promoter region (−1638 bp/0 bp) and (−1648 bp/0 bp). Assays were performed with the Dual Luciferase Reporter Assay Kit (Vazyme, Nanjing, China). Briefly, the firefly luciferase activity driven by the *TJP1* promoter was measured and normalized to the renilla luciferase activity from the PRL-TK plasmid to account for transfection efficiency and other experimental variations. The relative luciferase activity was calculated as the ratio of firefly luciferase activity to renilla luciferase activity.

### 2.6. Molecular Docking Analysis

Rigid protein–protein docking (ZDOCK) was conducted between ALKBH5 (PDB, ID: 4NJ4) and p65 (PDB, ID: 1MY5) to study the associations. The protein structural domain’s PDB format was obtained from the Protein Data Bank (http://www.rcsb.org/, accessed on 1 December 2024). The ZDOCK module was used to identify the docking sites and compute the ZDOCK scores.

### 2.7. Mouse Mastitis Model Construction

One male and two female mice were housed together and mated freely, and the females were separately housed after pregnancy. Female mice were separated from the infancy mice and were fasted and dehydrated for 2 h, 5–7 d after delivery. The mice were then intraperitoneally injected with either an IOX1 solution (14.182 mg/kg, dissolved in 5% DMSO), an equal volume of 5% DMSO, or PBS, with each mouse receiving a volume of 150 µL. Female mice were anesthetized and positioned supine, and the fourth pair of mammary glands and the surrounding skin were disinfected with 75% alcohol after 30 min. A blunt 1 mL syringe was used to inject pAKLUX1-*E. coli* saline suspension at 2 × 10^7^ CFU/mL of 50 μL into the mammary glands and the nipples were disinfected with iodine after the injection.

### 2.8. Mouse Live Imaging

Bioluminescent signals from *E. coli* were detected in mice using the small animal live imaging system (PerkinElmer, Waltham, MA, USA) while maintaining anesthesia with 1% isoflurane. Additionally, tomography was performed using different light wavelengths to construct three-dimensional models and fit the luminescence signals to the bones and some internal organs. Analyses were conducted with Living Image 4 (PerkinElmer, Waltham, MA, USA).

### 2.9. Transmission Electron Microscopy

Mice mammary tissues were washed with PBS, and the thinly sliced samples were fixed with 2.5% glutaraldehyde and stored at 4 °C. Ultrathin sections (70 nm) were cut using an ultramicrotome and stained with uranyl acetate and lead citrate. The samples were then observed via 100-KV H7650 TEM (Hitachi, Tokyo, Japan), and images were captured with an attached digital camera system.

### 2.10. Immunofluorescence Staining

MAC-T cells were cultured in 96-well plates (PerkinElmer, Waltham, MA, USA). The cells were fixed and permeabilized and then blocked with 1% BSA. The primary antibodies (p65 and TJP1) were diluted with PBS containing 1% BSA and incubated overnight at 4 °C. The cells were incubated with a mixture of green (Dylight 488) and yellow (Dylight 549) fluorescent secondary antibodies after washing with PBS. The cell nuclei were stained with DAPI (Beyotime, Shanghai, China). The cells were visualized with the PE Opera Phenix (PerkinElmer, Waltham, MA, USA). Table S2 presents the antibodies used.

### 2.11. HE and Immunohistochemistry (IHC) Staining

Mouse mammary tissue was isolated, fixed in 10% neutral buffered formalin, and processed into paraffin sections (5 µm). Sections were deparaffinized, rehydrated, and stained with hematoxylin and eosin (HE) for histological examination. For immunohistochemistry (IHC), sections were subjected to antigen retrieval in sodium citrate buffer (pH 6.0), blocked with 5% normal goat serum (Beyotime, Shanghai, China), and incubated with a primary antibody against p65 overnight at 4 °C. After washing, sections were incubated with a biotinylated secondary antibody and avidin–biotin peroxidase complex reagent, followed by DAB visualization and hematoxylin counterstaining. Images were captured using a light microscope (Zeiss, Oberkochen, Germany). Table S2 shows the antibody used.

### 2.12. RT-qPCR

RNA extraction and RT-qPCR methods were the same as previously described [17]. RNA extraction was performed using the TRIzol reagent (Invitrogen, Carlsbad, CA, USA) according to the manufacturer’s instructions. cDNA was synthesized from 1 µg of total RNA using the HiScript III RT SuperMix for qPCR (Vazyme, Nanjing, China), and qPCR was conducted using the AceQ qPCR SYBR Green Master Mix (Vazyme, Nanjing, China) on the ViiA 7 Real-Time PCR System (Applied Biosystems, Foster City, CA, USA). Gene expression levels were normalized to *ACTB*. Primer sequences used for qPCR are detailed in Table S3.

### 2.13. Western Blot

The Western blot method was the same as previously reported [17]. Briefly, proteins were separated by SDS-PAGE, transferred to PVDF membranes, and incubated with primary antibodies overnight at 4 °C. Membranes were then incubated with HRP-conjugated secondary antibodies for 1 h at room temperature. Signals were detected using an ECL substrate (Bio-Rad, Hercules, CA, USA) and imaged with a Tanon Imaging System (Tanon, Shanghai, China). Table S2 shows the antibodies used.

### 2.14. Statistical Analysis

Data were statistically analyzed using GraphPad Prism 9.0 (GraphPad Software, San Diego, CA, USA). For qPCR and Western blot experiments, significant differences between groups were assessed using Student’s *t*-tests for comparisons between two groups. For comparisons involving multiple groups, one-way ANOVA was performed. For the dual-luciferase assay, differences between groups were analyzed using Student’s *t*-tests. Data are presented as mean ± standard deviation (SD). A *p*-value of 0.05 was deemed statistically significant.

## 3. Results

### 3.1. ALKBH5 Inhibits E. coli-Induced Bovine Mammary Epithelial Cell Tight Junction Disruption

**Figure 1:**
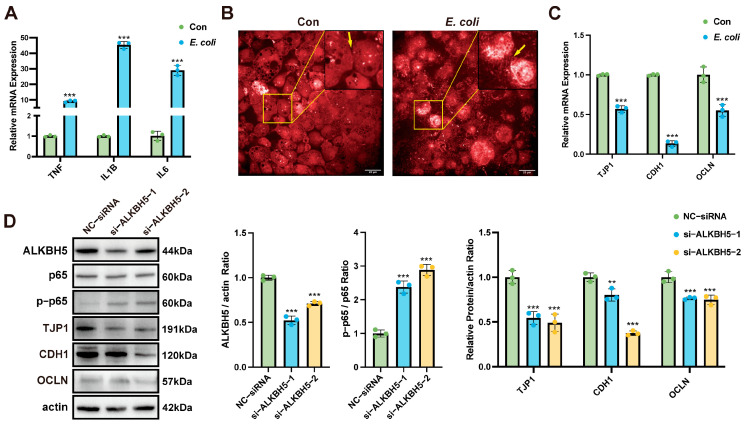
ALKBH5 inhibits *E. coli*-induced MAC-T cell tight junction disruption. (**A**) *E. coli* induced upregulation of *TNF*, *IL1B*, and *IL6* mRNA expressions in MAC-T cells (24 h infection). Measured by RT-qPCR, normalized to *ACTB*; (**B**) *E. coli* induced tight junction disruption in MAC-T cells (24 h infection), yellow arrows indicate inter-cellular junction. Scale bar = 20 µm; (**C**) *E. coli*-induced down-regulation of *TJP1*, *CDH1*, and *OCLN* mRNA expressions in MAC-T cells (24 h infection). Measured by RT-qPCR, normalized to *ACTB*; (**D**) ALKBH5 knockdown induced p65 phosphorylation and inhibited TJP1, CDH1, and OCLN protein expressions. MAC-T cells transfected with ALKBH5 siRNA (24 h) followed by *E. coli* infection (24 h). Analyzed by Western blotting, with actin as loading control. Data are mean ± SD (n = 3 replicates per group, 3 independent experiments). ** *p* < 0.01, *** *p* < 0.001.

*E. coli* induced a significant up-regulation of inflammatory factors *TNF*, *IL1B*, and *IL6* in bovine mammary epithelial cells (Figure 1A), indicating the activation of an acute inflammatory response. In addition, *E. coli* caused massive shedding of MAC-T cells and disrupted the cell tight junctions (Figure 1B), while down-regulating the mRNA expressions of tight junction-associated genes *TJP1*, *CDH1*, and *OCLN* (Figure 1C). These findings suggest that *E. coli*-induced inflammation compromises the integrity of the mammary epithelial barrier. To investigate the role of ALKBH5 in this process, we knocked down ALKBH5 using two siRNAs (Figure 1D). The results of Western blot showed that ALKBH5 knockdown activated the NF-κB signaling pathway, significantly inhibiting the expressions of TJP1, CDH1, and OCLN, with TJP1 expression being the most down-regulated (Figure 1D). These results suggest that ALKBH5 may inhibit *E. coli*-induced tight junction disruption by regulating the NF-κB/p65 pathway.

### 3.2. ALKBH5 Mediates p65 to Promote TJP1 Expression

**Figure 2:**
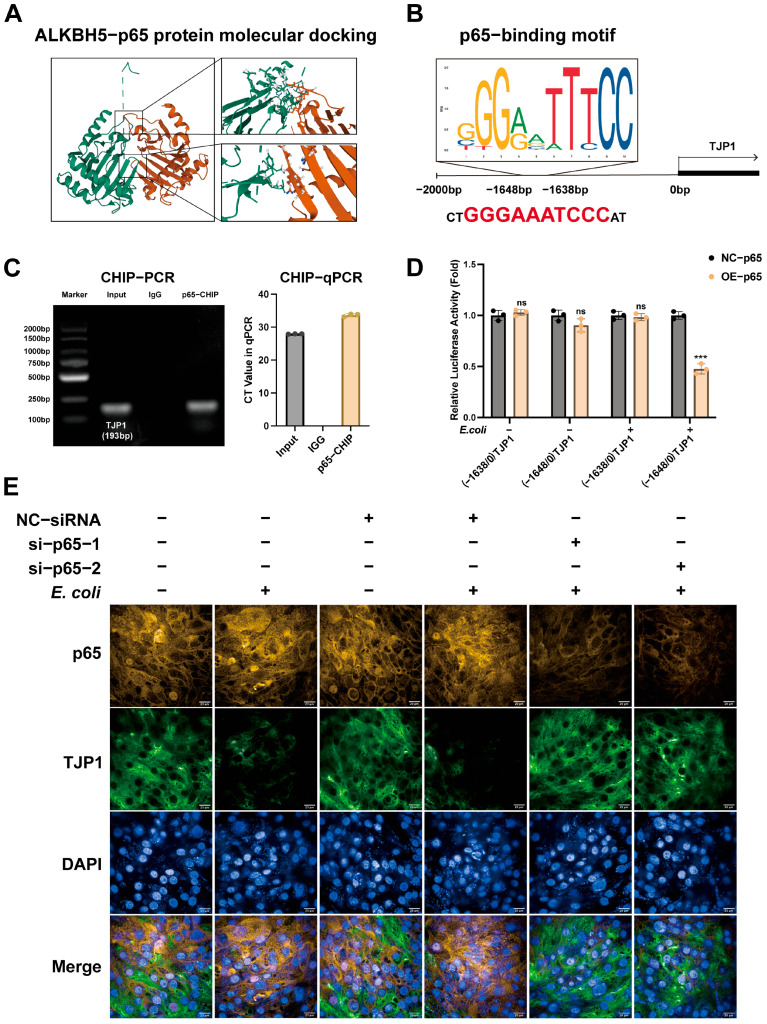
ALKBH5 mediates p65 to promote TJP1 expression. (**A**) Molecular docking analysis indicates that ALKBH5 binds to the p65 protein; (**B**) The DNA recognition motif of p65 perfectly matches part of the *TJP1* promoter; (**C**) CHIP-PCR/qPCR confirmed that p65 binds to the *TJP1* promoter; (**D**) Dual luciferase assays demonstrated that *E. coli* mediates p65 phosphorylation and translocation into the nucleus to inhibit the promoter activity of *TJP1*; (**E**) p65 inhibits TJP1 expression in MAC-T cells infected with *E. coli* for 24 h. Immunofluorescence staining shows p65 (yellow) and TJP1 (green) with DAPI (blue) indicating nuclei. Scale bar = 20 µm. Data are presented as mean ± SD (n = 3 replicates per group, 3 independent experiments), ns = no significant difference, *** *p* < 0.001.

P65 is crucial in the NF-κB pathway, significantly affecting *E. coli*-induced inflammation. To explore the regulatory mechanisms of *E. coli*-induced inflammation and tight junction disruption, we investigated the interaction of ALKBH5 with p65 and TJP1. Molecular interactions showed that ALKBH5 strongly interacted with p65 at the protein level, with a ZDOCK score of 928.558 (Figure 2A), suggesting that ALKBH5 may bind directly to p65. To further elucidate the effect of this interaction on TJP1, we found that the DNA binding motifs of p65 perfectly matched the −1648 bp/−1638 bp region of the *TJP1* gene by motif prediction analysis (Figure 2B). CHIP-PCR/qPCR confirmed that p65 bound to this region of the *TJP1* promoter (Figure 2C). Furthermore, dual-luciferase assays exhibited that p65 bound to the −1648 bp/−1638 bp region on the *TJP1* promoter as a transcription factor and regulated promoter activity. *E. coli* stimulated the NF-κB signaling pathway to be activated, which caused p65 phosphorylation to p-p65 and its translocation into the nucleus, inhibiting the promoter activity of *TJP1*, and thereby suppressing TJP1 expression (Figure 2D). Confocal microscopy observation further revealed that *E. coli* inhibited TJP1 expression, whereas p65 knockdown restored TJP1 expression (Figure 2E). Collectively, these results suggest that ALKBH5 mediates p65 to promote TJP1 expression, thereby promoting bovine mammary epithelial cell tight junctions.

### 3.3. E. coli Induces Mouse Mastitis and Disrupts Epithelial Cell Tight Junctions

**Figure 3:**
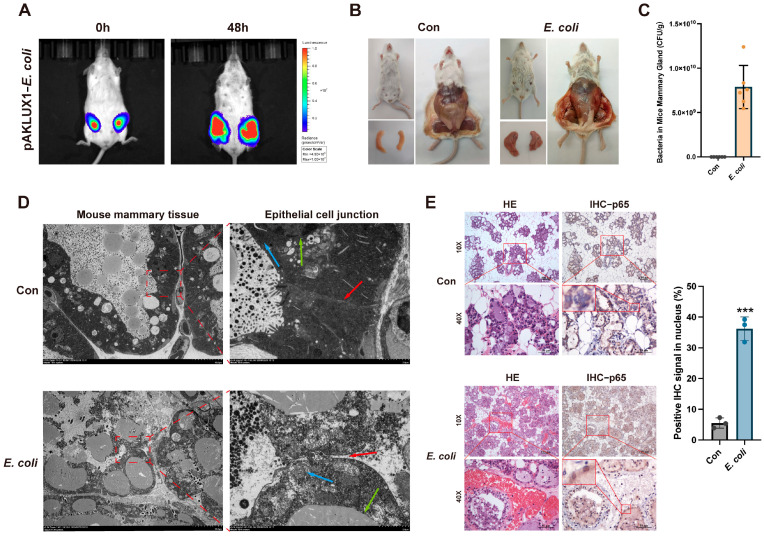
*E. coli* induces mice mastitis and disrupts epithelial cell tight junctions. (**A**) Small animal live imaging demonstrating the proliferation and spread of bioluminescent *E. coli* in the mammary glands of mouse; (**B**) Appearance, dissection, and mammary tissue of control and *E. coli*-infected mouse; (**C**) Bacterial load measurement in mammary tissue of control and *E. coli*-infected mouse. Data are presented as median bacterial load (CFU/g); (**D**) TEM observation of the damage of mouse mammary gland epithelial cell junction. Blue arrows indicate mitochondria, green arrows indicate nucleus, and red arrows indicate epithelial junction structures. Scale bar = 2 µm; (**E**) HE and IHC detection of mammary tissue of control and *E. coli*-infected mouse, and quantification of p65 nuclear translocation by IHC. Scale bar = 50 µm. Data are presented as mean ± SD (n = 6 mice per group), *** *p* < 0.001.

As ALKBH5 is highly conserved in eukaryotes [23], we further investigated the disruption of tight junctions in bovine mammary epithelial cells using a mouse mastitis model. To establish the mouse mastitis model, bioluminescent *E. coli* was used (Figure S1). Behavioral differences in mice due to mastitis were observed using the open field test (Figure S2), indicating the adverse effects of *E. coli* infection on the overall health of mice. *E. coli* proliferated abundantly in the mammary tissue and the infected area was expanded (Figure 3A). Dissection revealed that *E. coli* group mice had enlarged nipples and red, swollen mammary tissues (Figure 3B). Bacterial load measurement further confirmed that *E. coli* multiplied abundantly in mammary tissue, with a high median *E. coli* load of 7.405 × 10^9^ CFU/g (Figure 3C). TEM revealed that the control group had clear mammary gland structures with tight cell junctions, whereas the *E. coli* group exhibited swollen mitochondria, lysed nucleus, and disrupted cellular tight junctions (Figure 3D). HE staining indicated that the mammary gland structure in the *E. coli* group was disrupted, with epithelial cell detachment, hemorrhage, and inflammatory cell infiltration compared to the control group (Figure 3E). These findings confirm that *E. coli* induced acute mouse mastitis, characterized by significant tissue damage and inflammation. Further, IHC demonstrated that *E. coli* induced the translocation of p65 from the cytoplasm to the nucleus in epithelial cells, with an increase of approximately 30% (Figure 3E). These results suggest that *E. coli*-induced mastitis compromises the integrity of the epithelial barrier and promotes the nuclear translocation of p65.

### 3.4. Inhibition of ALKBH5 Aggravates Mastitis

**Figure 4:**
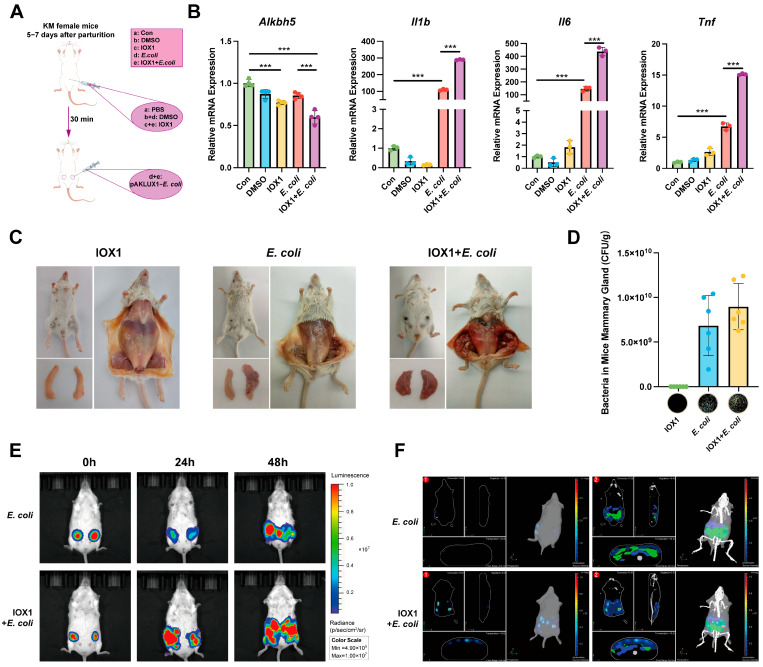
Inhibition of ALKBH5 aggravates mastitis. (**A**) Construction and grouping of the mouse mastitis model. Mice were intraperitoneally injected with ALKBH5 inhibitor IOX1 or controls (5%DMSO or PBS). Mammary glands were injected with bioluminescent *E. coli* after 30 min; (**B**) RT-qPCR showed that IOX1 inhibited *Alkbh5* expression and promoted *E. coli*-induced inflammatory cytokine expressions (*Il1b*, *Il6*, *Tnf*) in mouse mammary tissues. Data are normalized to *Actb*; (**C**) Dissection of mammary glands showing exacerbated tissue injury in mice treated with IOX1 + *E. coli* compared to *E. coli* alone; (**D**) Bacterial load measurement in mammary tissue showing higher *E. coli* concentrations in the IOX1 + *E. coli* group. Data are presented as median bacterial load (CFU/g); (**E**) Small animal live imaging demonstrating that IOX1 enhanced *E. coli* proliferation and spread in the mouse mammary glands. Luminescence signal intensity indicated bacterial load; (**F**) Three-dimensional modeling showed that IOX1 aggravated *E. coli* proliferation. Data are presented as mean ± SD (n = 6 mice per group), *** *p* < 0.001.

To investigate the effect of ALKBH5 in mastitis, mice were injected intraperitoneally with the ALKBH5 inhibitor IOX1 and controls received DMSO or PBS. The mammary glands were injected with bioluminescent *E. coli* after 0.5 h and assayed (Figure 4A). IOX1 significantly inhibited the expression of *Alkbh5* in mice, confirming the efficacy of the inhibitor. In addition, IOX1 treatment further exacerbated the *E. coli*-induced up-regulation of *Il1b*, *Il6,* and *Tnf* in mouse mammary tissues, suggesting that inhibition of ALKBH5 exacerbated the *E. coli*-induced inflammatory response (Figure 4B). The dissection results showed no injury to mammary tissue by IOX1, but mice in the IOX1 + *E. coli* group exhibited more severe mammary injury than the *E. coli* group (Figure 4C). Additionally, bacterial load measurements showed higher *E. coli* concentrations in mammary tissue in the IOX1 + *E. coli* group, suggesting that ALKBH5 inhibition promotes bacterial proliferation (Figure 4D). Small animal live imaging further demonstrated that IOX1-mediated ALKBH5 inhibition promoted *E. coli* proliferation, increased the infection area, and thus exacerbated mastitis (Figure 4E,F).

### 3.5. Inhibition of ALKBH5 Aggravates Epithelial Cell Tight Junctions Disruption

**Figure 5:**
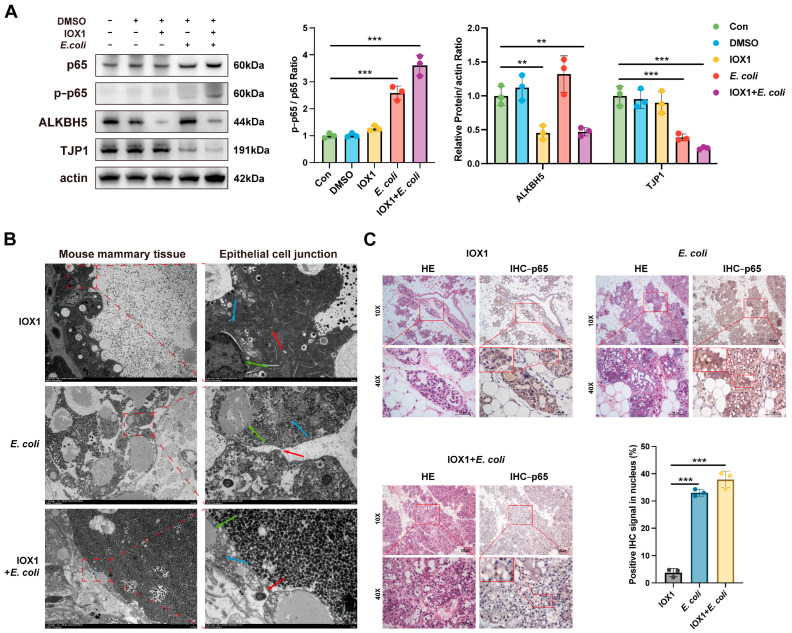
Inhibition of ALKBH5 aggravates epithelial cell tight junction disruption. (**A**) WB detected up-regulation of ALKBH5 and p-p65, and down-regulation of TJP1 in mouse mammary tissues during IOX1 induction. β-actin was used as a loading control; (**B**) TEM imaging of mammary glands of mice in the IOX1 group, the *E. coli* group, and the IOX1 + *E. coli* group. Blue arrows indicate mitochondria, green arrows indicate nucleus, and red arrows indicate epithelial junction structures. Scale bar = 2 µm; (**C**) HE and detection of mouse mammary tissue from the IOX1, *E. coli*, and IOX1 + *E. coli* groups, and quantification of p65 nuclear translocation by IHC. Scale bar = 50 µm. Data are presented as mean ± SD (n = 6 mice per group), ** *p* < 0.01, *** *p* < 0.001.

The effect of ALKBH5 inhibition on cell tight junctions was further explored. Western blot results showed that IOX1 markedly inhibited ALKBH5 expression in mouse mammary tissues and significantly enhanced *E. coli*-induced activation of the NF-κB signaling pathway and down-regulation of TJP1 expression (Figure 5A). TEM revealed cell tight junctions in the mammary tissue of the IOX1 group. In contrast, the *E. coli* group exhibited swollen nuclei and disrupted cell junctions. However, in the IOX1 + *E. coli* group, epithelial cells were shed with completely destroyed cell junctions (Figure 5B). These observations indicate that ALKBH5 inhibition not only exacerbates *E. coli*-induced inflammation but also severely compromises the structural integrity of the mammary epithelial barrier. In addition, compared to the IOX1 and the *E. coli* groups, the IOX1 + *E. coli* group exhibited more mammary epithelial cell detachment as well as p65 nuclear translocation (Figure 5C). This study revealed that ALKBH5 inhibition demonstrated less effect on the mammary gland in the physiological state but intensified *E. coli*-induced inflammation and epithelial cell tight junction disruption.

## 4. Discussion

Epidemiological investigations revealed that *E. coli* is one of the most prevalent pathogenic microorganisms in the clinical mastitis of cows, which poses a great danger to the dairy farming industry [24]. *E. coli* contains LPS on its surface as a Gram-negative bacterium, which activates the cellular innate immune response [25]. Heat-inactivated *E. coli* was used in this study to induce inflammatory injury in bovine mammary epithelial cells. Heat inactivation effectively preserved the bacterial antigen and avoided the experimental errors caused by bacterial proliferation. Additionally, another study confirmed that heat-inactivated *E. coli* retains important antigens, such as LPS, and is an excellent experimental material for constructing mastitis models [26,27].

Researchers have examined the mammary tissue of cows with mastitis, categorizing the lesions as mixed, lymphoplasmacytic, septic, necrotizing, and granulomatous [28]. Among these, *E. coli* contains high endotoxin levels, and its infection causes acute inflammatory injury, which is mainly characterized by acute necrotic lesions in the mammary gland [29]. *E. coli* colonized heavily and lesions, such as vascular damage, tissue edema, hemorrhage, and thrombosis, were seen in the mammary tissue in the breast of cows with clinical mastitis, with multiple areas of necrosis in the mammary parenchyma, as well as large neutrophil infiltration and abundant fibrin [30]. Our mouse mastitis model revealed histopathological features similar to those of clinical mastitis cows, including swelling and necrosis of mammary tissue and damage to epithelial cells.

Inflammation disrupts the epithelial barrier and cell tight junctions through the activation of multiple signaling pathways, which in turn affects TJP1 expression and localization, disrupting intercellular adhesion and barrier integrity [31,32]. Some severe inflammatory diseases cause locally intense immune responses, resulting in several life-threatening syndromes [33,34], which begin with altered permeability between epithelial cells and tight junction disruption [35,36]. Additionally, this study revealed that *E. coli*-induced inflammation disrupts epithelial cell tight junctions mainly through the NF-κB/p65 pathway. P65 plays a crucial role in the mechanism of cellular inflammatory injury due to infectious diseases. Research indicates that p65 contributes to heightened epithelial cell permeability in gastroenteritis, intensified by IL6 and TNF release, thereby further compromising the intestinal barrier [37]. Interleukins activate *TNF* gene expression via NF-κB, thereby significantly decreasing OCLN and TJP1 levels in bronchial mucosal epithelial cells [38]. *E. coli* infection in mice disrupts the mammary epithelial barrier and suppresses tight junction protein expression, such as TJP1 [39]. This study similarly revealed that *E. coli* induced p65 phosphorylation and reduced TJP1 expression, thereby disrupting cell tight junctions.

M^6^A has been demonstrated to affect various physiological and pathological processes, such as cell tight junctions [40]. This study used IOX1 as an ALKBH5 inhibitor [41,42], which further intensified *E. coli*-induced disruption of epithelial cell tight junctions, promoted *E. coli* proliferation, and eventually intensified inflammatory injury in mice. One study revealed that the bacterial load and pro-inflammatory cytokine levels were higher in the peritoneal cavity and blood of septic mice lacking ALKBH5 [43]. Some researchers revealed that it inhibited IL17A secretion by CD4^+^ T cells and the tropism of Th17 cells toward the site of inflammation using IOX1 to inhibit the expression of ALKBH5, which in turn inhibited inflammation [44]. Another study concluded that IOX1 intensified hyperoxia-induced lung bronchial injury in mice [45]. This study revealed that IOX1 inhibited ALKBH5 and intensified inflammation. IOX1 may inhibit immune cell production and migration, but it may exacerbate inflammatory injury by promoting cytokine production by worsening oxidative stress.

Studies focusing on ALKBH5 regulation of cell tight junctions and epithelial barrier protection are largely absent. In this study, ALKBH5 was confirmed to significantly inhibit p65 phosphorylation and nuclear translocation in inflammatory responses, thereby upregulating epithelial cell tight junctions. The failure to enter the nucleus blocked the transcription of its downstream genes, such as various inflammatory factors [46], causing an anti-inflammatory effect since the nuclear translocation of p65 is the essential basis for its function. Interestingly, this study further demonstrated the mechanism by which p65 phosphorylation regulates the *TJP1* promoter, which provides a deeper understanding of the association between cell inflammatory damage and tight junctions. Moreover, ALKBH5 may be a promising target for the treatment of mastitis and warrants further research to develop products.

## 5. Conclusions

**Figure 6:**
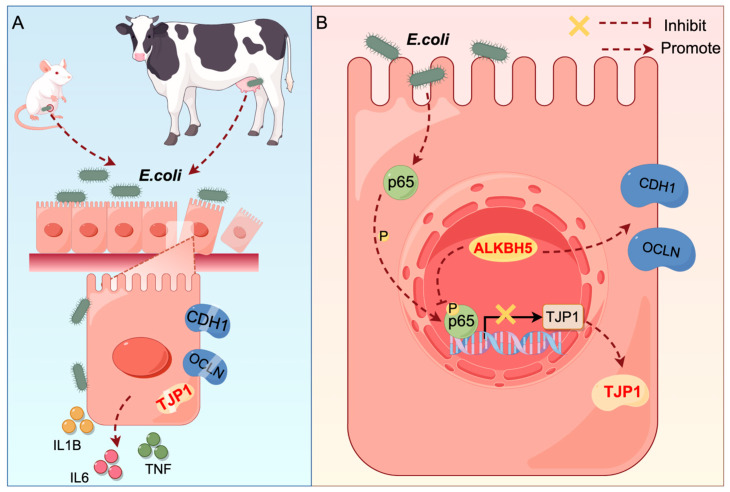
Schematic diagram of ALKBH5 protecting cell tight junction. (**A**) *E. coli*-induced mastitis disrupts epithelial cell tight junction; (**B**) ALKBH5 protects epithelial cell tight junction.

This study revealed that *E. coli*-induced acute mastitis disrupts the epithelial cell tight junction (Figure 6A). ALKBH5 inhibits inflammatory responses through the NF-κB/p65 signaling pathway, mediates TJP1 expression regulation by p65, and consequently improves cell tight junction (Figure 6B).

## Data Availability

All data generated or analyzed in this study are included in this published article. Data are available upon request from the corresponding author.

## Supplementary Materials

The following supporting information can be downloaded at https://www.mdpi.com/article/10.3390/cells14070521/s1, Table S1. The siRNA sequences used for gene knockdown, Table S2. Antibodies for WB, IHC, IF, and CHIP, Table S3. Sequence of primers used in qPCR, Figure S1. Construction and validation of bioluminescent *E. coli*, Figure S2. *E. coli* infection inhibits mice’s spontaneous activity.

## Abbreviations

*E. coli*	*Escherichia coli*
ALKBH5	AlkB Homolog 5
NF-κB	nuclear factor kappa-B
M^6^A	N^6^-methyladenosine
TJP1	tight junction protein 1
CDH1	cadherin 1
OCLN	occluding
TNF	tumor necrosis factor
IL1B	interleukin 1 beta
IL6	interleukin 6
ACTB	actin beta
TEM	Transmission Electron Microscope
IHC	Immunohistochemistry
IF	Immunofluorescence
CHIP	Chromatin Immunoprecipitation
