# Supplementary material for: ALKBH5 Improves the Epithelial Cell Tight Junctions to Inhibit Escherichia coli-Induced Mastitis
Source: Cells. 2025 Apr 1;14(7):521. doi: 10.3390/cells14070521 (PMC11988031; doi:10.3390/cells14070521)
Supplement: Supplementary file 1 [file cells-14-00521-s001.zip › Supplementary Materials.pdf]

Table S1. The siRNA sequences used for gene knockdown

siRNA	siRNA sequence（5′−3′）
si−ALKBH5−1	GCUGCAAGUUCCAGUUCAATT
si−ALKBH5−2	GCGCCGUCAUCAACGACUATT
si−p65−1	GCAGUUUGAUACCGAUGAA
si−p65−2	GGACGUACGAGACCUUCAA
NC−siRNA	UUCUCCGAACGUGUCACGUTT

Table S2. Antibodies for WB, IHC, IF, and CHIP

Antibodies	Purpose	Source
actin	WB	bs-0061R, Bioss, China
ALKBH5	WB	bs-20540R, Bioss, China
TJP1	WB, IF	1G4A1, proteintech, China
NF−κB p65	IHC, IF, CHIP	8242S, CST, US
NF−κB p65	WB	bs-20355R, Bioss, China
Phospho−NF−κB p65	WB	3033T, CST, US
CDH1	WB	bs-10009R, Bioss, China
OCLN	WB	bs-1495R, Bioss, China
HRP, Goat Anti−Rabbit IgG	WB	A21020, Abbkine, US
HRP, Goat Anti−Mouse IgG	WB	A21010, Abbkine, US
Dylight 488, Goat Anti−Mouse IgG	IF	A23210, Abbkine, US
Dylight 549, Goat Anti−Rabbit IgG	IF	A23320, Abbkine, US

Table S3. Sequence of primers used in qPCR

Gene	Primers sequence（5′−3′）	
ACTB	F: AGATCAAGATCATCGCGCCC	R: TAACGCAGCTAACAGTCCGC
TNF	F: TCTTCTCAAGCCTCAAGTAACAAGC	R: CCATGAGGGCATTGGCATAC
IL1B	F: TTCCATATTCCTCTTGGGGTAGA	R: AAATGAACCGAGAAGTGGTGTT
IL6	F: CAGCAGGTCAGTGTTTGTGG	R: CTGGGTTCAATCAGGCGAT
CDH1	F: GCCCAGCCGAGAACTTTCAG	R: CCACCATCACTCCCTGATGT
OCLN	F: CTTTCAAAAAGGGCTCCCGC	R: TGGATATTCCCTGATCCAGTCG
TJP1	F: CAGCCCAGAGAGACACCAC	R: AGCAAAACCCTCTCGTAGGC
ALKBH5	F: CCCATCCACATCTTCGAGCG	R: AGCAGCGTATCCACTGAGCAC

F: forward, R: reverse

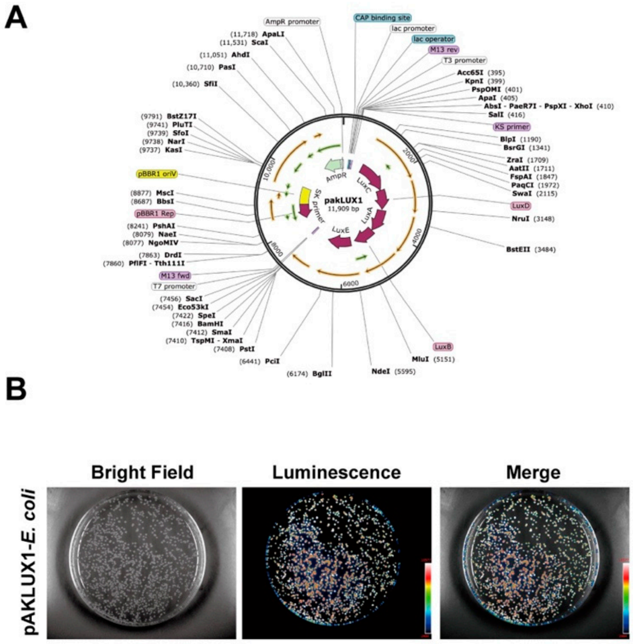

Figure S1 Construction and validation of bioluminescent *E. coli*

(A) Schematic structure of pAKLUX1 plasmid; (B) Chemiluminescence imaging system to detect luminescence signals of recombinant strains. Bright Field for bright field shots, Luminescence for fluorescence shots, and Merge for synthetic images.

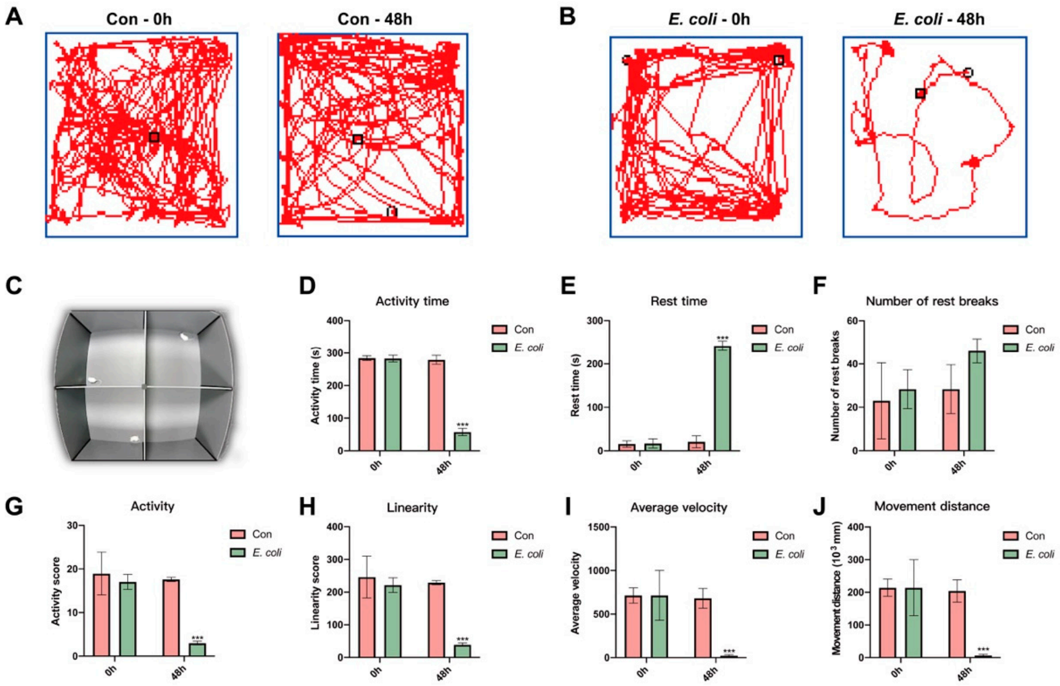

Figure S2 *E. coli* infection inhibits mice's spontaneous activity

(A) Spontaneous activity route recordings of control mice in open field over 5 min, with squares indicating the start recording position and circles indicating the end recording position; (B) Spontaneous activity route recordings of *E. coli*-infected mice in open field over 5 min; (C) Schematic map of the open field; (D) Open field experiments analyzed in the system to record the mice's time of activity, (E) resting time, (F) number of rests, (G) activity, (H) activity linearity, (I) average speed, and (J) total movement distance. Six mice were set up as biological replicates for each group, and each experiment was repeated 3 times. ANOVA test was used, ***p<0.001.
