# Supplementary figures and images for: ALKBH5 Improves the Epithelial Cell Tight Junctions to Inhibit Escherichia coli-Induced Mastitis
Source: Cells. 2025 Apr 1;14(7):521. doi: 10.3390/cells14070521 (PMC11988031; doi:10.3390/cells14070521)

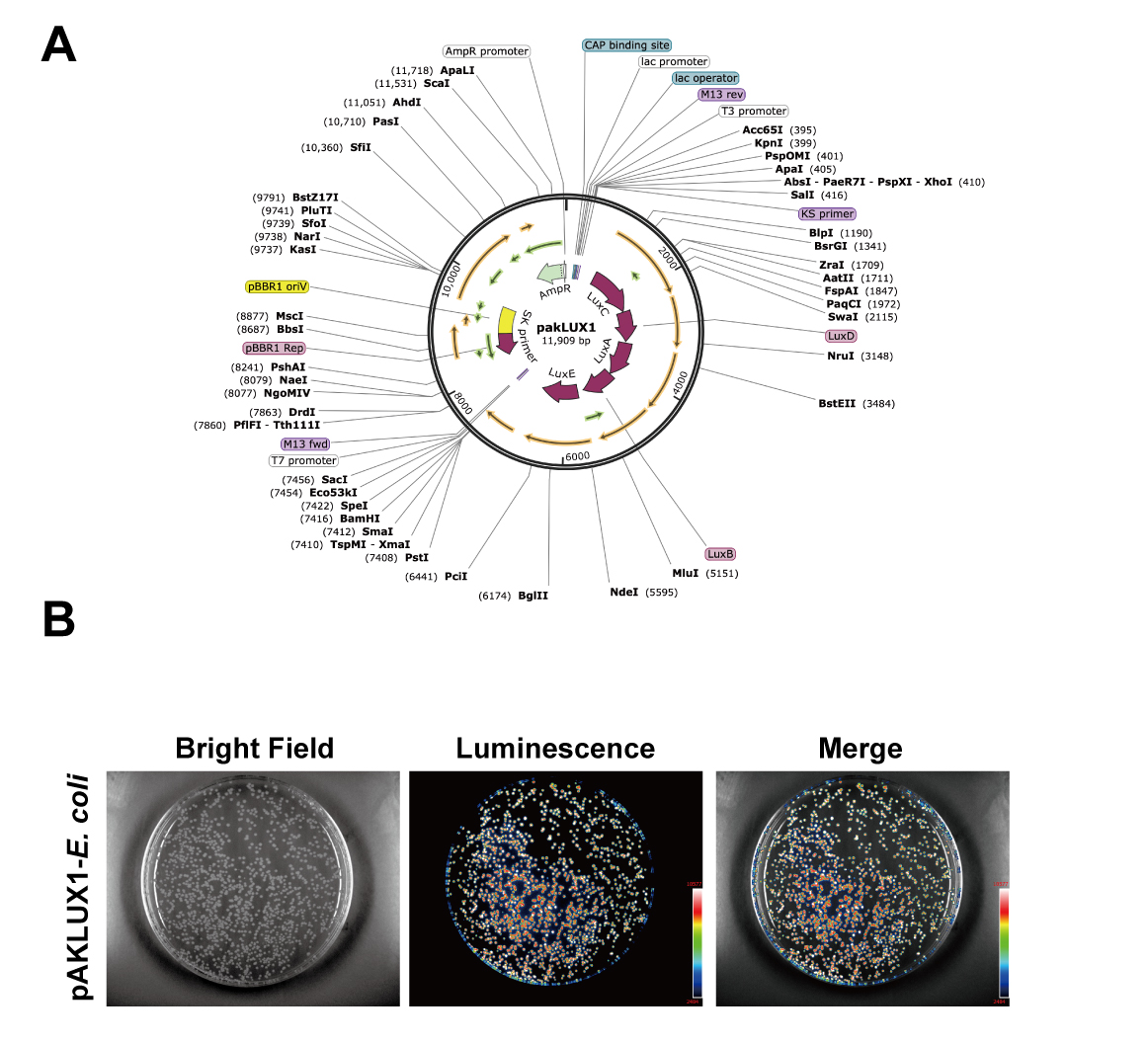

Supplement: Supplementary file 1 [file cells-14-00521-s001.zip › Fig.S1 Construction and validation of bioluminescent E. coli.png]

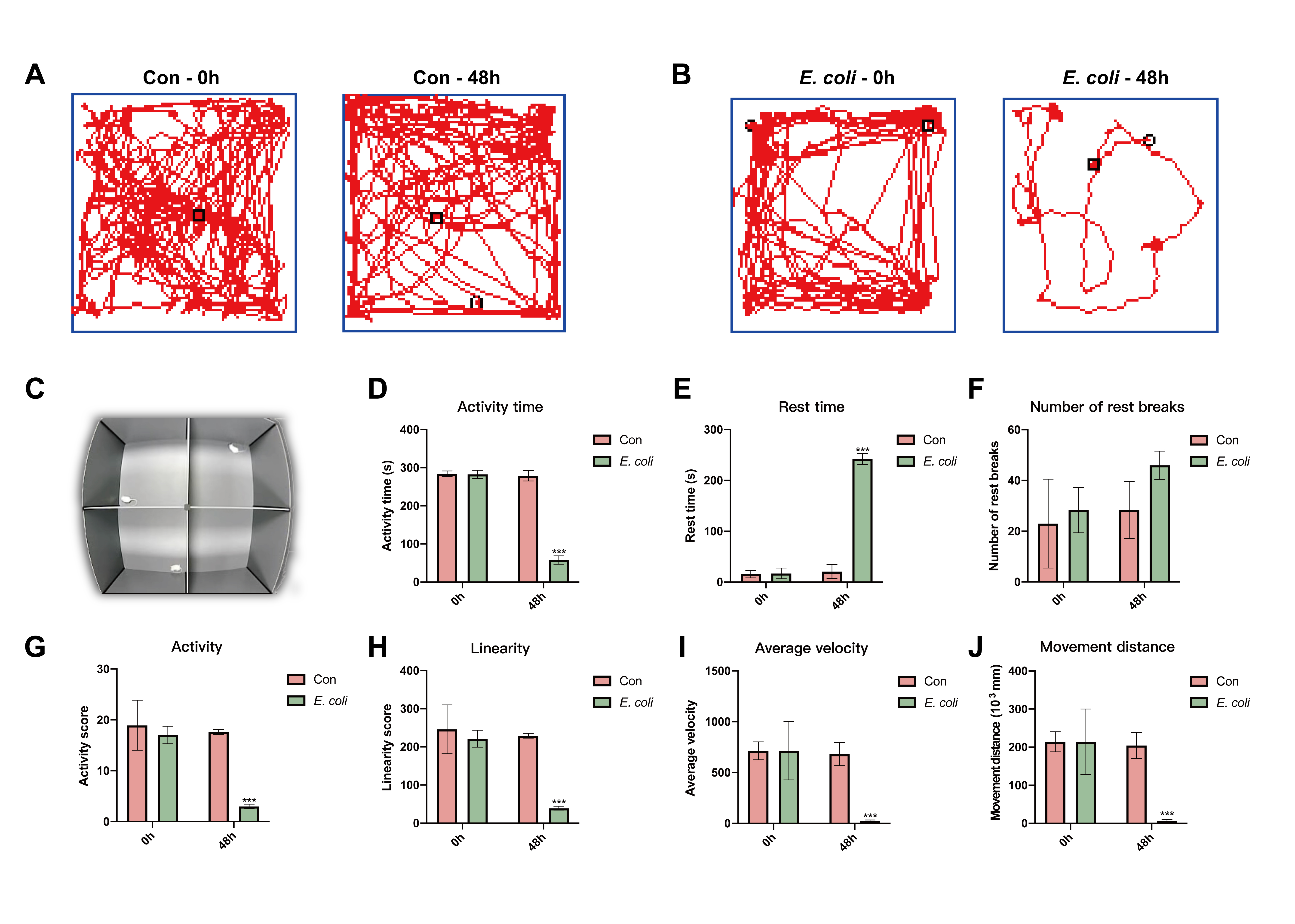

Supplement: Supplementary file 1 [file cells-14-00521-s001.zip › Fig.S2 E. coli infection inhibits mice's spontaneous activity.png]
